# Supplementary material for: 12-Lipoxygenase inhibition delays onset of autoimmune diabetes in human gene replacement mice
Source: JCI Insight. 2024 Dec 20;9(24):e185299. doi: 10.1172/jci.insight.185299 (PMC11665553; doi:10.1172/jci.insight.185299)
Supplement: Supplemental data [file jciinsight-9-185299-s070.pdf]

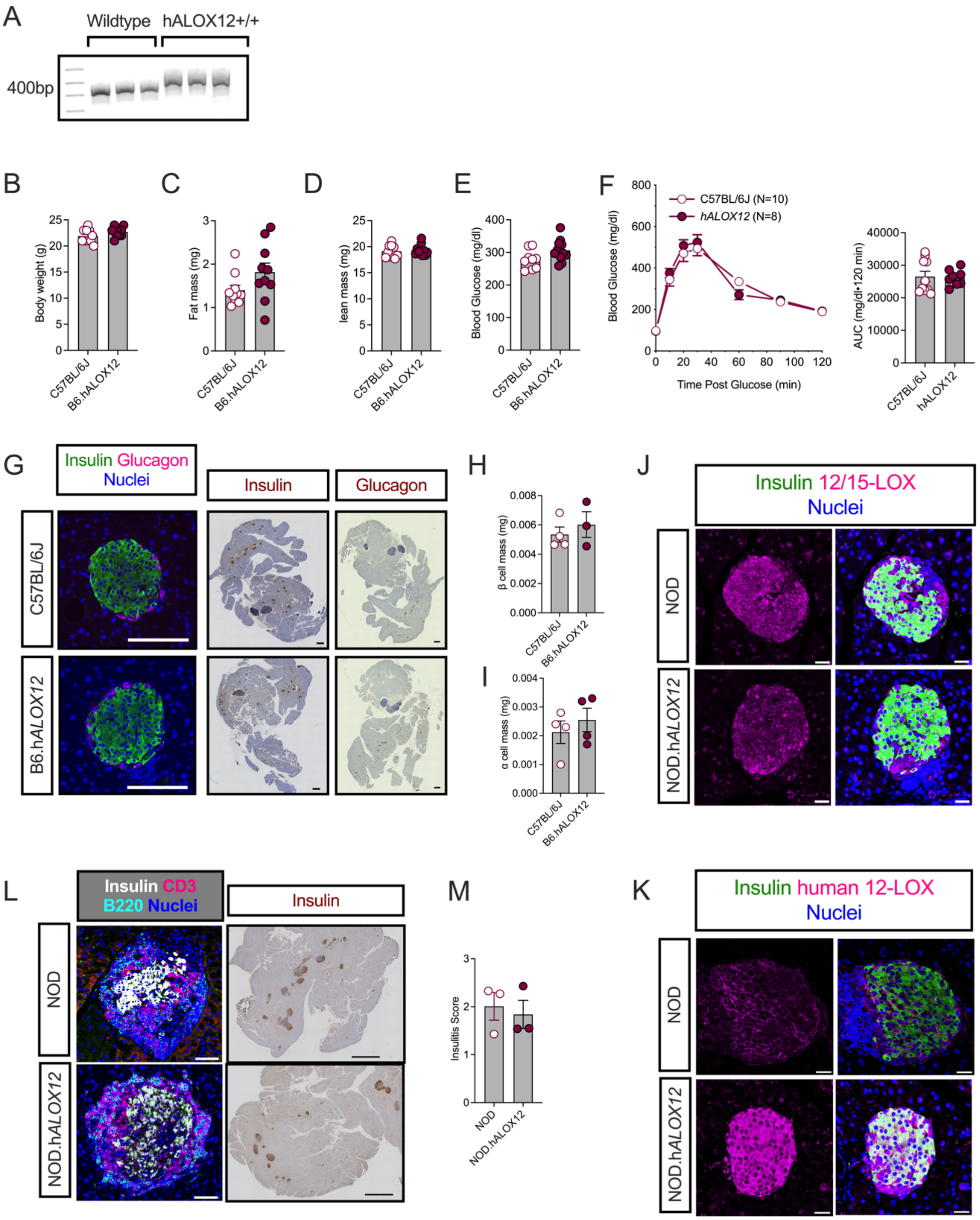

Supplemental Figure 1: Comparison of *B6.hALOX12* to *C57BL/6J* mice and *NOD.hALOX12* to *NOD.ShiltJ* mice at 10 weeks of age. (**A**) Genotyping results of *B6.hALOX12* mice. (**B**) Body weight. (**C**) Fat mass. (**D**) Lean mass. (**E**) Random-fed blood glucose. (**F**) IPGTT and AUC (*right panel*) of 8 week old male *B6.hALOX12* mice (N=8) compared to *C57BL/6J* mice (N=10). (**G**) Pancreata from the mice indicated stained for glucagon (magenta), insulin (green) and nuclei (blue) (left panels); insulin (brown, middle panels); or glucagon (brown, right panels). Scale bars = 50 μm. (**H**) β cell mass, each dot

represents an individual mouse. (**I**) α cell mass, each dot represents an individual mouse. (**J**) Pancreata from female *NOD* and *NOD.hALOX12* mice stained for 12/15-LOX (magenta), insulin (green), and nuclei (blue) (left panels). Scale bars = 20 μm. (**K**) Pancreata from female *NOD* and *NOD.hALOX12* mice stained for 12-LOX (magenta), insulin (green), and nuclei (blue) (left panels). Scale bars = 20 μm. (**L**) Pancreata from female *NOD* and *NOD.hALOX12* mice stained for CD3 (magenta), B220 (teal), insulin (white), and nuclei (blue) (left panels) or insulin (brown, right panels). Scale bars = 50 μm. (**M**) Insulitis score, each dot represents data from an individual mouse. Data are presented as mean ±SEM and statistical significance was determined by a two-tailed T-test.

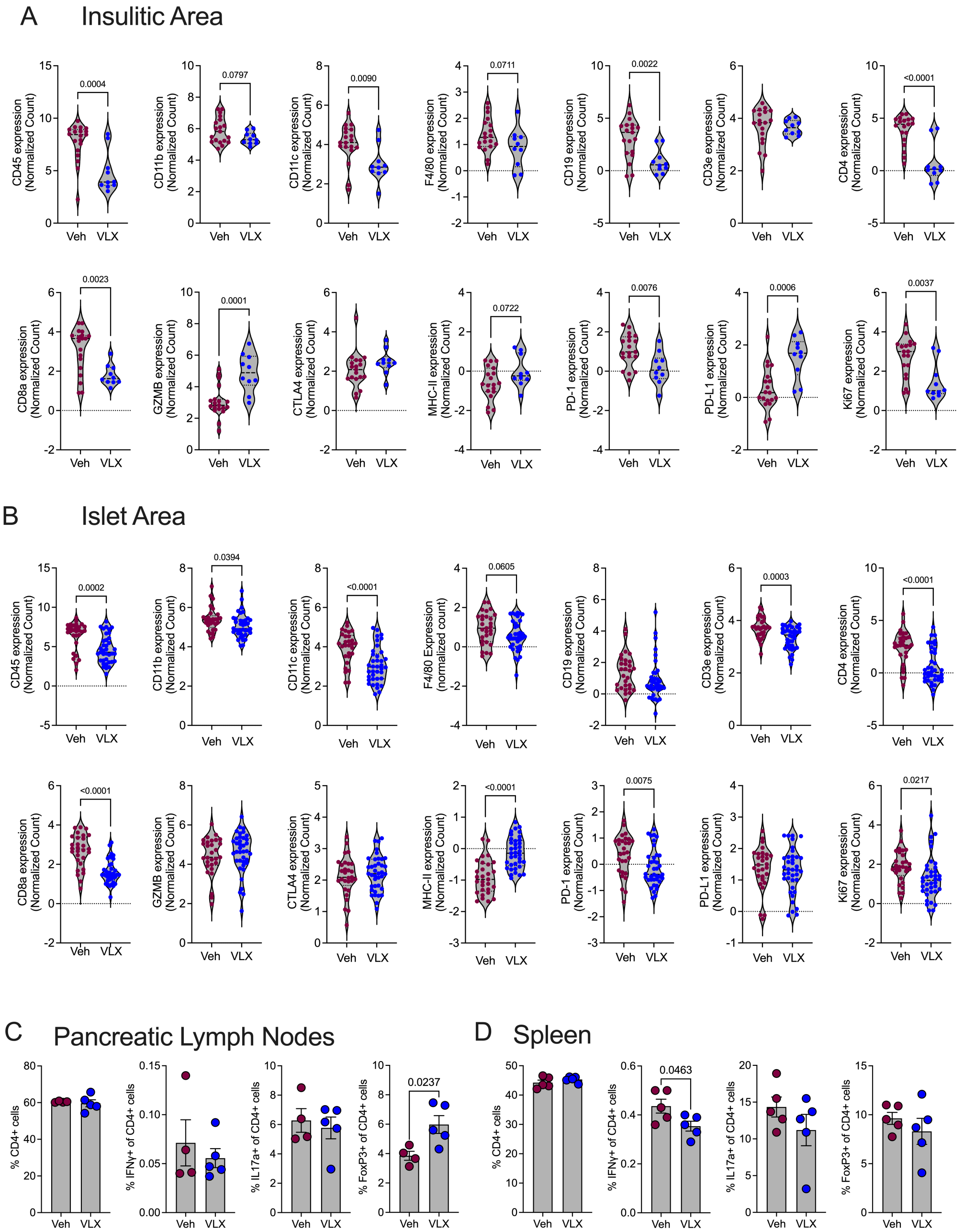

Supplemental Figure 2: Spatial proteomics analysis and flow cytometry analysis of *NOD.hALOX12* mice treated with vehicle or VLX-1005. Female *NOD.hALOX12* mice were treated for 4 weeks with either vehicle or VLX-1005. Nanostring® spatial proteomics were performed in the pancreas in regions of interest that included the peri-islet insulitic area (**A**) or the intra-islet area (**B**) for the indicated markers. Each dot represents a different region of interest. Pancreatic lymph nodes (**C**) or spleen (**D**) were isolated from treated mice and subjected to flow cytometry for the indicated markers. Each dot represents data from a single mouse. Data are presented as mean ±SEM and statistical analysis was performed using a two-tailed T-test.

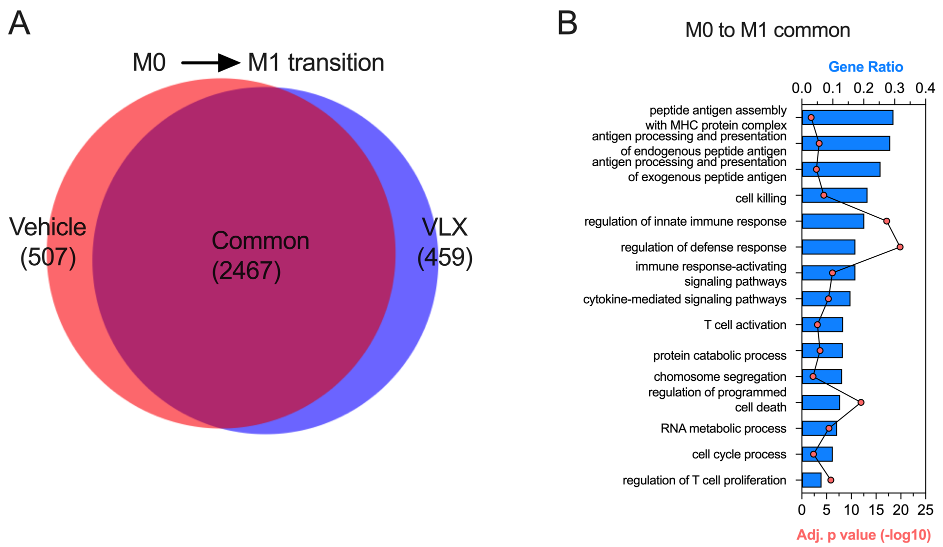

Supplemental Figure 3: RNA-sequencing analysis of M1-like bone marrow derived macrophages. Bone marrow-derived macrophage (BMDMs) were isolated and polarized to the M1-like state and treated with vehicle or VLX-1005 (10 µM) during polarization. RNA was isolated and sequenced. (**A**) Differential gene expression identified between vehicle- and VLX-1005-treated macrophages during the M0 to M1 transition phase. (**B**) Gene ontology pathway analysis of the common differentially expressed genes.

Supplemental Table 2: Lipidomics results of non-12-lipoxygenase products from serum of mice treated with vehicle or VLX-1005 for 1 weeks. Data are presented as mean ±SEM and statistical analysis was performed using a two-tailed T-test.

	Vehicle	30 mg/kg VLX-1005 SDD	p-value
**5-HETE**	96.1± 35.3	16.0 ± 2.5	0.053
**11(12)-EET**	18.1 ± 8.1	2.2 ± 0.4	0.083
**14,15-DHET**	12.6 ± 4.4	5.4 ± 0.7	0.141
**11,12-DHET**	6.4 ± 2.3	4.0 ± 0.5	0.350
**14(15)-EET**	16.1 ± 7.8	3.6 ± 0.6	0.110
**8(9)-EET**	24.8 ± 11.1	3.8 ± 1.0	0.096
**5(6)-EET**	140.7 ± 55.3	24.2 ± 5.3	0.070
**18-HETE**	3.0 ± 1.5	1.7 ± 0.4	0.433
**TX-B2**	12.4 ± 2.7	5.5 ± 2.2	0.086
**6-keto-PGF1a**	39.2 ± 11.8	1.1 ± 0.6	0.012
**Leukotriene-B4**	1.5 ± 0.7	0.1 ± 0.1	0.080
**5-oxo-ETE**	12.2 ± 3.7	3.9 ± 0.8	0.058
**13-HDHA**	19.1 ± 7.0	3.4 ± 0.9	0.056
